# Long‐term remission and survival in dogs with high‐grade, B cell lymphoma treated with chemotherapy with or without sequential low‐dose rate half‐body irradiation

**DOI:** 10.1111/jvim.16840

**Published:** 2023-09-12

**Authors:** Matthew P. Best, Rod C. Straw, Elias Gumpel, Darren R. Fry

**Affiliations:** ^1^ Eastcott Referrals Swindon United Kingdom; ^2^ Brisbane Veterinary Specialist Centre Brisbane Queensland Australia; ^3^ The Australian Animal Cancer Foundation Albany Creek Queensland Australia; ^4^ Small Animal Specialist Hospital North Ryde New South Wales Australia

**Keywords:** CHOP, controlled, cure, radiation therapy

## Abstract

**Background:**

Standard of care for dogs with high‐grade lymphoma, multiagent chemotherapy, achieves good initial responses but long‐term remissions are infrequent; previous studies using half‐body irradiation suggest improved long‐term outcomes.

**Hypothesis:**

The addition of low‐dose rate half‐body irradiation would improve outcomes in dogs with B‐cell lymphoma.

**Animals:**

Client‐owned dogs with stage III or higher, substage a, B‐cell lymphoma that achieved complete remission after 4 doses of multiagent chemotherapy.

**Methods:**

A case‐controlled design comparing 2‐year remission and survival rates between dogs treated with CHOP‐based chemotherapy and those treated with chemotherapy and sequential low‐dose rate half‐body irradiation.

**Results:**

Thirty‐eight dogs were enrolled with 18 included in final analysis, 9 prospectively‐enrolled dogs and 9 case‐matched historical controls. The irradiation cohort's 2‐year disease‐free rate was 56% whereas median duration exceeded the 730‐day study period compared with 0% and 261 days in the chemotherapy only group. Remission duration significantly differed between cohorts (*P* < .01), hazard ratio 0.218 (95% CI: 0.06‐0.77). The irradiation cohort's 2‐year survival rate was 78% with median overall survival duration exceeding the 730 day study period compared with 11% and 286 days in the chemotherapy only group. Overall survival time significantly differed between cohorts (*P* < .02), hazard ratio 0.173 (95% CI: 0.03‐0.839).

**Conclusions and Clinical Importance:**

The improved long‐term outcome achieved by dogs administered sequential low‐dose rate half‐body irradiation in this study is similar to previous observational studies. Where long‐term remission is sought in dogs with B‐cell lymphoma low‐dose rate half‐body irradiation could be considered in addition to standard chemotherapy.

**Abbreviations**
DFI: disease‐free interval
LDR‐HBI: low dose‐rate half‐body irradiation
OS: overall survival

## 1 INTRODUCTION

Multicentric lymphoma in dogs is the most common hematopoietic cancer of dogs and the current standard of care is multiagent chemotherapy, which includes doxorubicin.^1^ Many chemotherapy protocols have been proposed over the past decades, but outcomes remain broadly similar with remission rates of 80% to 90%, duration of remission of 9 to 10 months, and long‐term (>2 years) remission rates of just 5% to 10%.^1^, ^2^, ^3^, ^4^, ^5^ Advances in human oncology have resulted in significant improvements in long‐term (>5 years) remission rates and most notably the addition of the monoclonal antibody rituximab to standard chemotherapy protocols has resulted in 60% long‐term remission rates.^6^, ^7^, ^8^ In veterinary oncology a number of treatments have been investigated or proposed as potential adjunctive therapeutics that might produce a similar improvement in the long‐term outcome for dogs with lymphoma receiving multiagent chemotherapy protocols^9^; these include immunotherapy,^10^, ^11^, ^12^ bone marrow ablation with autologous hematopoietic stem cell transplantation,^13^, ^14^ and half‐body irradiation.^15^, ^16^, ^17^, ^18^, ^19^, ^20^, ^21^, ^22^, ^23^ To date, all these modalities have shown some promise but additional studies to characterize their efficacy are lacking and none have been adopted as the standard of care. Of these proposed adjunctive therapies, half‐body irradiation has the largest number of studies investigating its usage. The rapid increase in the availability of dedicated veterinary linear accelerators provides the potential for widespread adoption of this therapy if a safe and efficacious protocol could be demonstrated.

Irradiation is highly cytotoxic to malignant lymphocytes^24^, ^25^ exerting effects through both DNA damage and induction of apoptosis.^26^, ^27^ The potential adverse effects of wide field irradiation have reduced the uptake in dogs but the recent adoption of sequential low dose‐rate half‐body irradiation (LDR‐HBI) has reduced adverse effects, thus benefiting dogs, but also importantly allowing a shorter interval between radiation fractions.^21^, ^22^, ^23^ Administration of LDR‐HBI to dogs in remission with multicentric lymphoma produces long‐term remission rates of 42% to 64%,^21^, ^22^, ^23^ similar to outcomes in human oncology with rituximab and chemotherapy. It remains to be shown that this result can be repeated in other centers and in a case‐controlled study.

Our hypothesis is that dogs with multicentric lymphoma which achieve complete remission will have improved long‐term remission rate (>2 years) when treated with chemotherapy and LDR‐HBI compared with a case‐controlled individuals treated with chemotherapy only. A secondary measure of survival rate to 2 years is also considered to ensure that any effect of LDR‐HBI on survival was also assessed.

## 2 MATERIALS AND METHODS

### 2.1 Inclusion criteria

Privately‐owned dogs with naturally occurring lymphoma treated between 1 November 2011 and 31 October 2018 were included in the study. All dogs had to have a confident cytological or histological diagnosis of intermediate or large cell lymphoma with compatible clinical signs, be stage III or higher, be substage a, be immunophenotyped as B cell lymphoma via immunohistochemistry, immunocytochemistry, flow cytometry or PCR for antigen receptor rearrangement and be clinically in remission after 1 cycle of the chemotherapy protocol outlined in Table 1. Written owner consent was an enrolment criterion for all dogs in the study patient group. Staging and general health/comorbidity screening was at the discretion of the clinician in charge of the case taking into account clinical findings, financial constraints, and owner preferences. Dogs were excluded if they had a known comorbidity likely to limit life‐expectancy to <12 months or if there was any clinical or pathological suspicion of indolent lymphoma.

**TABLE 1:** Treatment protocol.

Protocol	Week																		
	1	2	3	4	5	6	7	8	9	10	11	12	13	14	15	16	17	18	19
Study group																			
Vincristine (0.7 mg/m^2^ IV)	•		•						•		•			•		•			
Cyclophosphamide (250 mg/m^2^ PO over 3 days)		•								•					•				
Doxorubicin (30 mg/m^2^ IV)				•								•					•		
L‐Asparaginase (10 000 u/m^2^ up to 10 000 u SC)							•												
LDR‐HBI cranial body						•													
LDR‐HBI caudal body								•											
Control group																			
Vincristine (0.7 mg/m^2^ IV)	•		•			•		•			•		•			•		•	
Cyclophosphamide (250 mg/m^2^ PO over 3 days)		•					•					•					•		
Doxorubicin (30 mg/m^2^ IV)				•					•					•					•

### 2.2 Study dogs

Study dogs that met the above criteria were prospectively enrolled between November 2016 and October 2018 where owners elected to include LDR‐HBI in their protocol. The treatment protocol was followed as per Table 1 with treatment delays if dogs had a neutrophil count of <1.5 × 10^9^/L, a platelet count of <75 × 10^12^/L or if they were clinically unwell. Dose delay durations, dose adjustments remained at the discretion of the clinician treating the case whereby delays and adjustments could be recommended with higher neutrophil or platelet counts based on prior experience with each dog or level of client risk aversion.

Dogs were heavily sedated or anesthetized in the week preceding the first dose of radiation to allow dog positioning for radiation planning. Radiation delivery was manually calculated using body separation measurements, as previously described.^22^ During the establishment of this treatment at this center a CT was performed on the first dog with 3‐dimensional planning to verify dosimetry using Pinnacle version 9.10 (Philips Radiation Oncology Systems, Fitchburg, Wisconsin) as per the method described by Laing et al.^15^ Close correlation was found with the manual calculation and manual calculations were used for subsequent dogs. For planning and therapy, dogs were placed in a large Perspex tray to allow consistent positioning and dogs were marked to ensure consistency. Dogs were anesthetized and irradiated using a Siemens Mevatron MXE (Siemens, Germany) in their tray on the floor, producing an increased source to surface distance with a median of 212.9 cm (range, 208.1‐215.9) with the increased source‐to‐surface distance meaning the machine delivered rate of 200 cGy/min was reduced to deliver low‐dose rate administration, with each fraction delivered over ~15 minutes. A total of 6 Gy of low‐dose‐rate irradiation was administered at a rate of 40 to 50 cGy/min and these doses were administered equally divided between left and right lateral recumbency to ensure homogenous dose distribution. While this dose rate is higher than previously published^21^, ^22^ personal communication with the primary author of these publications had suggested a dose rate of 40 to 50 cGy/min was similarly tolerated. Radiation was administered first to the cranial half of the body and then to the caudal half 2 weeks later. Note that the inclusion of LDR‐HBI was offered to all dogs with lymphoma presenting to the study hospital and meeting the inclusion criteria outlined above between November 2016 and October 2018. Where inclusion criteria were met, including written owner consent, all dogs were included in the study.

### 2.3 Case‐control dogs

Dogs that met the above criteria between November 2011 and October 2016 were retrospectively selected as matched case controls as outlined below. Cases were identified via a search of practice software for all dogs administered vincristine during this period followed by manual examination of the records to assess inclusion and exclusion criteria. Note that during this period half‐body irradiation was not available at this hospital or elsewhere in the country. The treatment protocol was followed as per Table 1 with treatment delays if dogs had a neutrophil count of <1.5 × 10^9^/L, a platelet count of <75 × 10^12^/L or if they were clinically unwell. Dose delay durations and dose adjustments remained at the discretion of the clinician treating the case whereby delays and adjustments could be recommended with higher neutrophil or platelet counts based on prior experience with each dog or level of client risk aversion.

### 2.4 Case matching

All study dogs were enrolled in the study where inclusion criteria were met, regardless of treatment and clinical outcomes. An independent board‐certified oncologist was presented with anonymized age, sex, neuter status, breed, weight, and stage for all dogs and, without knowledge of the study, was asked to match each study dog with 1 control dog taking into account known prognostic criteria for lymphoma to produce the best available comparison for each dog. Only case‐matched dogs took further part in the study analysis.

### 2.5 Data collection

Data on adverse effects from LDR‐HBI was collected and scored via Veterinary Cooperative Oncology Group common terminology criteria for adverse events^28^ and dogs were followed for 2 years with disease‐free interval (DFI) and overall survival (OS) times obtained from owners and clinical notes at the study hospital and the dogs' primary veterinarians. Remission status was based on nonstandardized lymph node palpation and general physical examination in both cohorts with recheck frequency at the discretion of the clinician in each case.

### 2.6 Power analysis

A power analysis was performed during study design using Stata Statistical Software, release 15 (StataCorp LLC, College Station, Texas) based on data from previous studies using optimistic estimates of 2‐year disease free rate in chemotherapy‐treated dogs. This estimated that 25 dogs would be required in each group to achieve a power of 0.8 to detect a statistically significant difference at *P* < .05, where likelihood of remission at 2 years was 10% in the control group and 40% in the study group using a 1‐sided log‐rank analysis.

### 2.7 Statistical analysis

Disease free rate at 2 years and OS were compared with dogs censored if still in remission or alive at 730 days after the first dose of chemotherapy. Data were analyzed statistically using Stata Statistical Software, release 17 (StataCorp LLC, College Station, Texas). Cox regression analysis was performed to calculate hazard ratios with 95% confidence intervals. Kaplan‐Meier analysis was performed for both outcomes and these were further assessed with log‐rank analysis with statistical significance assessed via a Chi‐square test. In all analyses, *P* < .05 was considered statistically significant.

## 3 RESULTS

### 3.1 Dogs

Within the study group, 9 dogs were enrolled and included in the study analysis. Cases where owners elected not to consent to LDR‐HBI but otherwise met inclusion criteria were not recorded. Mean age at first dose of chemotherapy was 5.7 years (range 2‐10 years) while mean bodyweight was 30.2 kg (range 14‐65 kg). There were 4 neutered females, 4 neutered males and 1 entire male. Breeds included 4 crossbreeds, 2 Staffordshire Bull Terriers and 1 each of a Labrador Retriever, a Kelpie and a Golden Retriever. All dogs were staged as per WHO guidelines as a minimum of stage III, incomplete staging prevented specific designation.

A total of 29 dogs were included in the control group. Of these 9 were selected as matched controls for corresponding study dogs, as described in the methods section and these are described here, while the other 20 dogs were not included in further analysis. The mean age at the first dose of chemotherapy was 6.9 years (range 3‐11 years) while mean body weight was 27.7 kg (range 17‐50 kg). There were 4 neutered females and 5 neutered males. Breeds included 4 crossbreeds and 1 each of a Border Collie, Blue Heeler, Great Dane, Hungarian Vizsla, Labrador Retriever, and Whippet. Two dogs were staged as a III, 6 dogs were incompletely staged but were at least stage III and the remaining dog was stage IV.

### 3.2 Protocol completion and LDR‐HBI adverse effects

**FIGURE 1:**
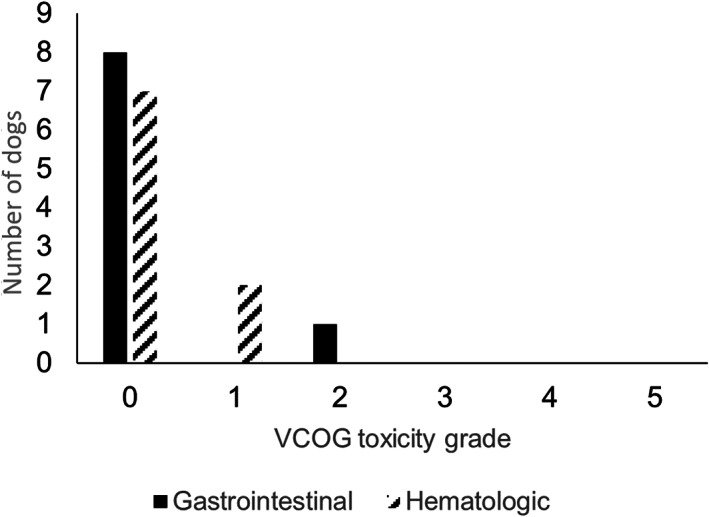
Observed adverse effects after cranial body low‐dose rate half‐body irradiation.

**FIGURE 2:**
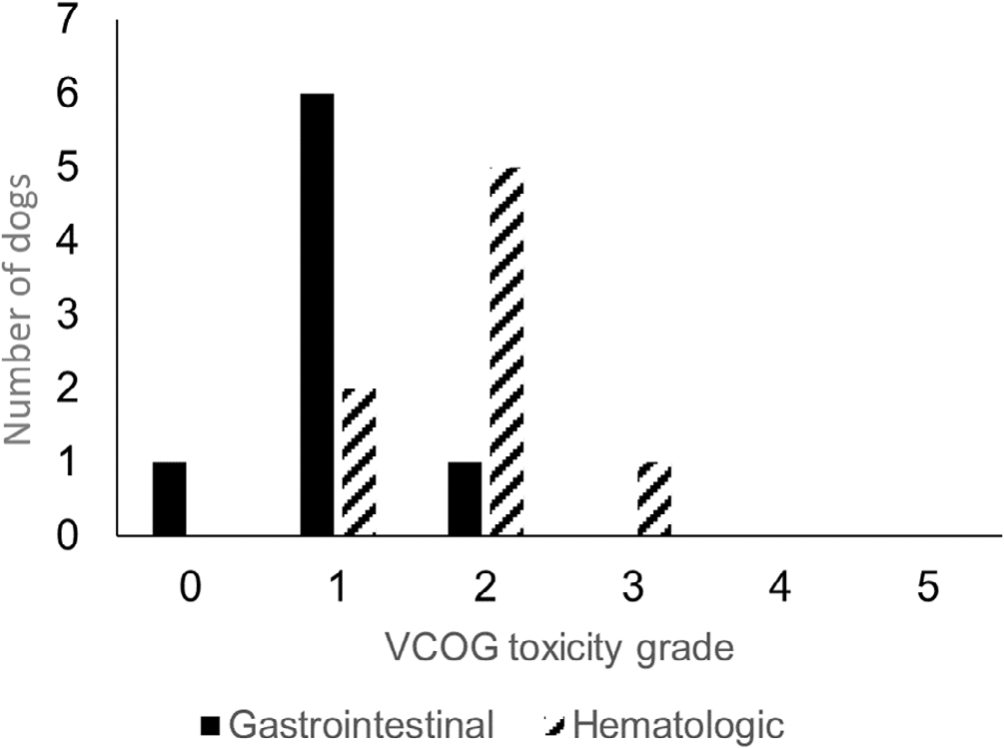
Observed adverse effects after caudal body low‐dose rate half‐body irradiation.

Two dogs, 1 from the study group and 1 from the control group, came out of remission before completing the chemotherapy protocol (124 and 126 days, respectively) while a third dog did not receive the caudal dose of LDR‐HBI and, while chemotherapy was continued, disease recurrence was noted at 103 days. The other 8 study dogs received both cranial and caudal LDR‐HBI. The adverse effects observed from the cranial and caudal LDR‐HBI are outlined in Figures 1 and 2. The dog that did not receive the second dose of LDR‐HBI had grade 2 gastrointestinal complications and grade 1 myelosuppression with the latter being prolonged and preventing administration of the second dose.

All dogs in the study and control groups had complete follow up.

### 3.3 Outcomes

Clinical impression of a strong clinical benefit to the inclusion of LDR‐HBI prompted an interim analysis before completion of the intended 25 case recruitment.

**FIGURE 3:**
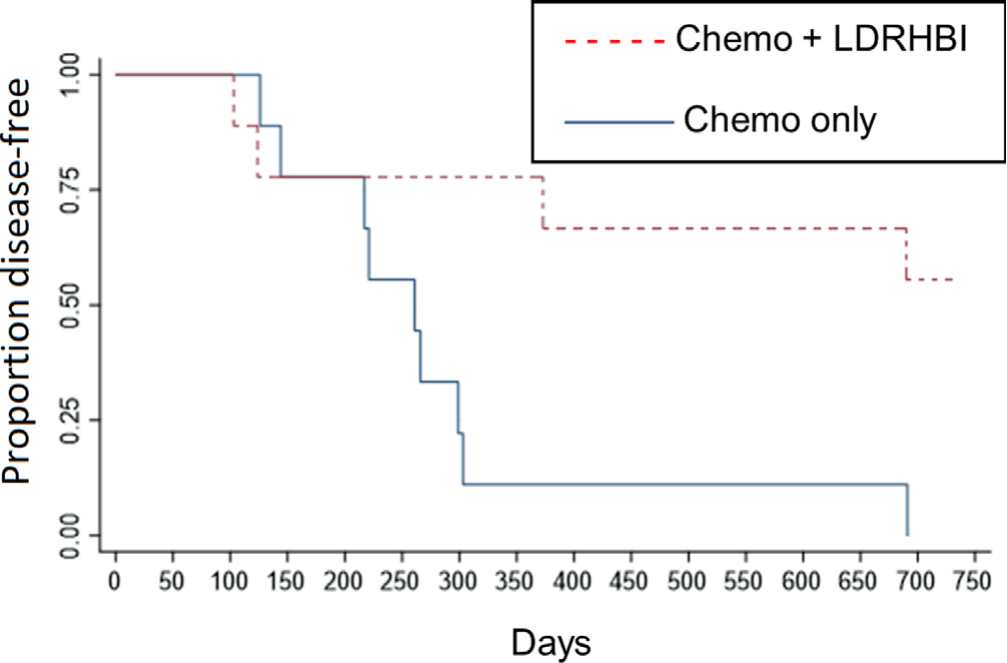
Remission KM curves. Kaplan‐Meier curves showing the proportion of dogs disease‐free for dogs treated with chemotherapy alone or chemotherapy and low‐dose rate half‐body irradiation. Median disease‐free interval was 261 days in the chemotherapy group and was not reached (>730 days) in the low‐dose rate half‐body irradiation with chemotherapy group, these outcomes were significantly different (*P* = .01).

Within the study group, 5 dogs (56%) were still in first remission at 730 days while within the control group no dogs (0%) were still in first remission at 730 days. The 5 dogs still in remission were censored at 730 days for DFI analysis. Kaplan‐Meier analysis is shown in Figure 3 and showed a median DFI of 261 days in the control group, while median DFI was not reached in the study group and this difference was statistically significant (*P* = .01). Hazard ratio was 0.218 (95% CI: 0.06‐0.77).

**FIGURE 4:**
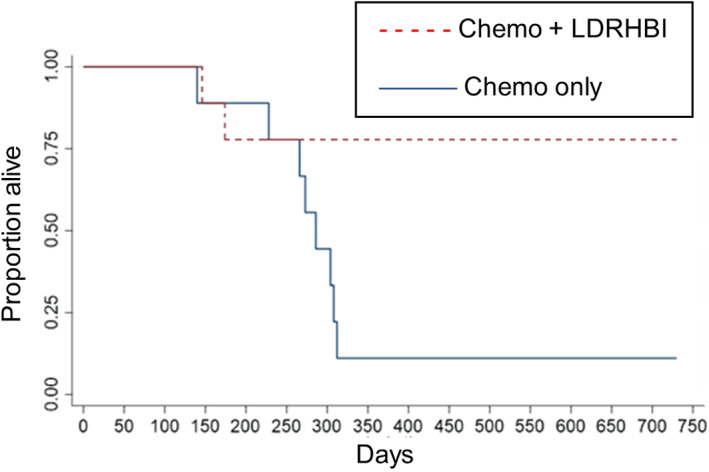
Survival KM curves. Kaplan‐Meier curves showing the proportion of dogs alive for dogs treated with chemotherapy alone or chemotherapy and low‐dose rate half‐body irradiation. Median overall survival was 286 days in the chemotherapy group and was not reached (>730 days) in the low‐dose rate half‐body irradiation with chemotherapy group, these outcomes were significantly different (*P* = .02).

Analysis of OS time showed 7 of 9 of the study dogs were still alive at 730 days, 2 of these having achieved a second remission with a second cycle of chemotherapy, while 2 had died. These 7 dogs were censored at 730 days for the analysis of OS. Within the control group, 1 of 9 was known to be alive and in a second remission after a second cycle of chemotherapy, 8 had died. This 1 dog was censored at 730 days for the analysis of OS. Kaplan‐Meier analysis is shown in Figure 4 and showed median OS of 286 days for the control group while median OS was not reached for the study group, this difference was statistically significant (*P* = .02). Hazard ratio was 0.173 (95% CI: 0.03‐0.839).

## 4 DISCUSSION

The treatment of lymphoma in dogs has long produced tension between the high rate of initial response and the low rate of long‐term remission. The low rate of long‐term remission is in contrast to the situation in human oncology and historically, owner expectations have been limited to the opportunity to delay disease progression. Our study showed a significantly increased rate of long‐term remission and OS in dogs receiving LDR‐HBI in addition to chemotherapy compared with dogs receiving chemotherapy alone. The 56% rate of long‐term remission observed in this study is similar to that seen in human patients with B cell lymphoma when treated with chemotherapy and immunotherapy and raises the possibility that it might be possible to switch from palliative chemotherapy to a curative intention when treating canine lymphoma. The protocol used was similar in duration to a standard chemotherapy protocol, was well‐tolerated, and could be adopted by any veterinary hospital with access to appropriate radiation therapy facilities and expertise.

Irradiation is highly lethal to malignant lymphocytes.^24^, ^25^ Lymphocyte death is achieved through standard mechanisms of radiation‐induced DNA‐damage, but also by the induction of cell death through activation of apoptotic pathways.^26^, ^27^ Furthermore, intrinsic and acquired mechanisms of cell resistance to chemotherapy and radiation are independent, producing the potential for an effective multimodal approach to therapy. In humans, radiation therapy remains an efficacious adjunct to chemotherapy and immunotherapy in non‐Hodgkin's lymphoma.^29^, ^30^ In dogs, adverse effects of wide‐field radiation include myelosuppression and gastrointestinal complications^15^ and this has led to the use of half‐body irradiation, where the treatment is divided into 2 fractions sequentially radiating each half of the body, allowing the recovery of healthy tissues between fractions. One challenge remains the potential that malignant lymphocytes could translocate from the unirradiated caudal body to the irradiated cranial body between fractions, which would undermine the value of treatment, and minimizing this translocation is a key therapeutic target in half‐body irradiation. The protocol employed in this study was selected with the intent to minimize the translocation of malignant cells using LDR‐HBI, treating dogs in complete remission and treating with anti‐neoplastic medications between LDR‐HBI fractions. The use of low‐dose rate irradiation reduces the myelosuppression experienced without significant reduction in lymphocyte mortality. The mechanism of this selectivity of low‐dose rate irradiation is likely the induction of apoptotic pathways selectively within lymphocytes.^26^, ^27^ The reduced myelosuppression encountered with LDR‐HBI allowed a short interdose interval and in this study most dogs had a 2‐week interval between doses of LDR‐HBI. The selection of dogs that were already in complete remission before the first fraction of LDR‐HBI meant dogs were likely to have a reduced tumor burden and residual cancer cells were less likely to be actively dividing or mobile within the body. The dogs were also administered L‐asparaginase between doses of LDR‐HBI. This medication was selected to inhibit malignant cell replication and movement during this critical period while avoiding the myelosuppressive effects which might be encountered with alternative chemotherapeutics which might add to the myelosuppression encountered from the LDR‐HBI. It should be noted that vincristine has been used for the same purpose in other studies with similar overall outcomes and adverse effects.^21^, ^22^

In line with previous studies, LDR‐HBI was generally well‐tolerated and in our study adverse effects were restricted to gastrointestinal and hematological complications. The cranial dose of LDR‐HBI was well‐tolerated with a low rate of mild to moderate adverse effects encountered. The caudal dose of LDR‐HBI was associated with a high rate of mild to moderate adverse effects but only 1 dog encountered a severe (Veterinary Cooperative Oncology Group—Common Terminology Criteria for Adverse Events grade 3) hematological adverse effect. While larger cohorts will be required to fully define any longer‐term adverse effects (>2 years) of LDR‐HBI and to uncover the frequency of more severe toxicity within this treatment group, these results suggest that overall this protocol is well‐tolerated.

One limitation of this study was the selection process for the control group with retrospective enrolment of the control group. This study design was selected to avoid the inherent bias of allowing owner choice to influence treatment modality as LDR‐HBI by using dogs for whom this treatment was not available. Prospective, blinded, randomized selection of treatment modality would be ideal but would be challenging to implement, most notably because of the financial differences between treatment modalities. A number of steps were taken to mitigate any bias introduced by this approach including: selection of dogs from the recent past; all controls were treated at the same hospital, by the same clinicians and using the same chemotherapy protocols as the study dogs; independent refinement of the eligible historic controls through individual case matching by a blinded and independent ECVIM‐CA boarded medical oncologist. The primary outcome of DFI was selected to reduce the impact of owner decision‐making, which can have a large impact on variables such as OS. Note that there was no change in recommended monitoring of remission status between control and study dogs, which was at the discretion of the clinicians, and that all dogs had complete follow up to either 2 years or death.

Another limitation was the small final sample size of 18 dogs. This number was less than that calculated in the power analysis because of the observation of marked increase in survival times in the study group which prompted an interim analysis showing marked, and statistically significant, differences in outcome between groups. While a false positive result must be carefully considered when using a small sample size, the presence of strong clinical and statistical differences between the groups and the closeness of the observed events in both cohorts to the outcomes achieved with similar protocols at other centers support the finding of a marked improvement in long term outcome in dogs treated with LDR‐HBI. The small sample size might also reduce the sensitivity for low‐incidence, but clinically significant, events, particularly adverse effects. Significant adverse effects were not observed in our cohort but larger studies would be required to assess for the incidence of infrequent but severe adverse effects.

Staging was always discussed with the owners but often not carried out so as not to divert potential funds where this might limit the owner's ability to complete a treatment protocol. As a result, most dogs have incomplete staging and in this study no dogs had complete staging, including bone marrow sampling. This prevents further investigation of the relationship between stage of lymphoma and the response to LDR‐HBI and represents a potentially interesting area for research in the future.

As part of our study, the study and control dogs had differences in their chemotherapy protocols including the study dogs receiving fewer doses of chemotherapy than their chemotherapy only counterparts but being administered L‐asparaginase, which was not administered to the chemotherapy only dogs. The addition of L‐asparaginase was to attempt to reduce the risk of malignant cell translocation between doses of LDR‐HBI as outlined above. The addition of L‐asparaginase to standard multiagent chemotherapy protocols has previously been studied^2^, ^31^ and there is no evidence that this addition impacts on long‐term outcome, and as such it is considered unlikely that the absence of L‐asparaginase in the protocols of control dogs contributed to their inferior outcome.

The final limitation of the study was that enrolment was limited to “substage a” dogs with B cell lymphoma that achieved complete remission after the first cycle of chemotherapy. These inclusion criteria were selected to allow clearer comparison of groups using the cohort previously suggested to show the best outcomes when LDR‐HBI was added to their treatment. Further studies investigating the potential benefits of this treatment in dogs with “substage b” lymphoma and T cell lymphoma would define whether this treatment would be beneficial in these cohorts.

In conclusion, our study provides further evidence that LDR‐HBI alongside multiagent chemotherapy results in significantly improved long‐term DFI and OS in dogs with multicentric B cell lymphoma who have achieved complete remission through initial chemotherapy dosing and does so with mild to moderate adverse effects in this cohort. The increasing availability of radiation therapy in veterinary practice opens the potential for this effective and reproducible treatment to become a standard treatment option for dogs with B cell lymphoma.

## CONFLICT OF INTEREST DECLARATION

Authors declare no conflict of interest.

## OFF‐LABEL ANTIMICROBIAL DECLARATION

Authors declare no off‐label use of antimicrobials.

## INSTITUTIONAL ANIMAL CARE AND USE COMMITTEE (IACUC) OR OTHER APPROVAL DECLARATION

Approved (CA 2016/10/1008) by the Animal Ethics Committee, DAF Queensland Government, Australia.

## HUMAN ETHICS APPROVAL DECLARATION

Authors declare human ethics approval was not needed for this study.
